# Maybe you can turn me on: CRISPRa-based strategies for therapeutic applications

**DOI:** 10.1007/s00018-022-04175-8

**Published:** 2022-02-12

**Authors:** Elvir Becirovic

**Affiliations:** Department of Pharmacy - Center for Drug Research, Ludwig-Maximilians-Universität München, Munich, Germany

**Keywords:** CRISPRa, CRISPR-mediated transcriptional activation, Gene activation, Transcriptional activation, Cas9-VPR, Cas9-SAM, Cas9-VP64, Cas9-MPH, Gene therapy

## Abstract

Since the revolutionary discovery of the CRISPR-Cas technology for programmable genome editing, its range of applications has been extended by multiple biotechnological tools that go far beyond its original function as “genetic scissors”. One of these further developments of the CRISPR-Cas system allows genes to be activated in a targeted and efficient manner. These gene-activating CRISPR-Cas modules (CRISPRa) are based on a programmable recruitment of transcription factors to specific loci and offer several key advantages that make them particularly attractive for therapeutic applications. These advantages include inter alia low off-target effects, independence of the target gene size as well as the potential to develop gene- and mutation-independent therapeutic strategies. Herein, I will give an overview on the currently available CRISPRa modules and discuss recent developments, future potentials and limitations of this approach with a focus on therapeutic applications and in vivo delivery.

## Main text

### CRISPR-Cas tools for therapy

The discovery of the CRISPR (clustered regularly interspaced short palindromic repeats)/Cas (CRISPR-associated protein) system has revolutionized the way genetic or acquired diseases can be treated. For its natural function in adaptive bacterial immune defense against viruses, the Cas enzyme requires CRISPR RNA (crRNA) that contains sequence segments from the viral genome responsible for binding to the target virus-derived DNA (spacer), and trans-activating crRNA (tracrRNA), which originates from the bacterial genome. tracrRNA and crRNA act together to recruit the Cas complex to the viral genome so that it can be cut and rendered harmless [1, 2]. For programmable manipulation of the bacterial and mammalian cell genomes, single guide (sg)RNA was developed by fusing the tracr and crRNA elements [1]. Since then, a wide range of different CRISPR-Cas derivatives were developed which differ in the species from which the Cas enzyme is derived, the architecture of the single guide RNAs, the number and type of proteins fused to Cas, or the endonuclease activity of the enzyme [3, 4]. Since its FDA-approval for investigational new drug application in 2019 for treatment of Leber congenital amaurosis, a severe form of retinal dystrophy caused by a mutation in the CEP290 gene (ClinicalTrials.gov Identifier: NCT03872479), the CRISPR-Cas technology has finally cleared the crucial regulatory hurdle for human application. Since then it has been applied in several clinical trials utilizing CRISPR-Cas to target genetic or acquired diseases (NCT04601051, NCT04191148, NCT03057912).

The currently available CRISPR-Cas modules can be used to excise or correct genome segments or to induce changes in the gene expression at specific loci [3, 4]. The gene transcription-modifying Cas modules typically contain the endonuclease-deficient Cas9 protein from *Streptococcus pyogenes* (*Sp*dCas9), to which functional domains capable of transcriptional activation (CRISPRa) or repression (CRISPRi) are fused. Endonuclease deficiency of Cas9 is usually achieved by introducing mutations in the corresponding functional domains of Cas9 [1, 5]. However, the enzyme also loses its nuclease activity in the presence of sgRNAs with truncated spacer sequences (< 16 bp) allowing the development of more sophisticated strategies for the treatment of specific diseases [6]. Transcriptional changes can e.g. be induced by recruitment of gene activating or repressive transcriptional factors or indirectly, i.e., by modifying the chromatin structure. While this review focuses on CRISPRa, it is worth noting here that the basic principles regarding the rational design of individual CRISPRa modules, their application spectra as well as their advantages and disadvantages largely apply to their CRISPRi counterparts.

### CRISPRa modules for epigenome editing

**Fig. 1:**
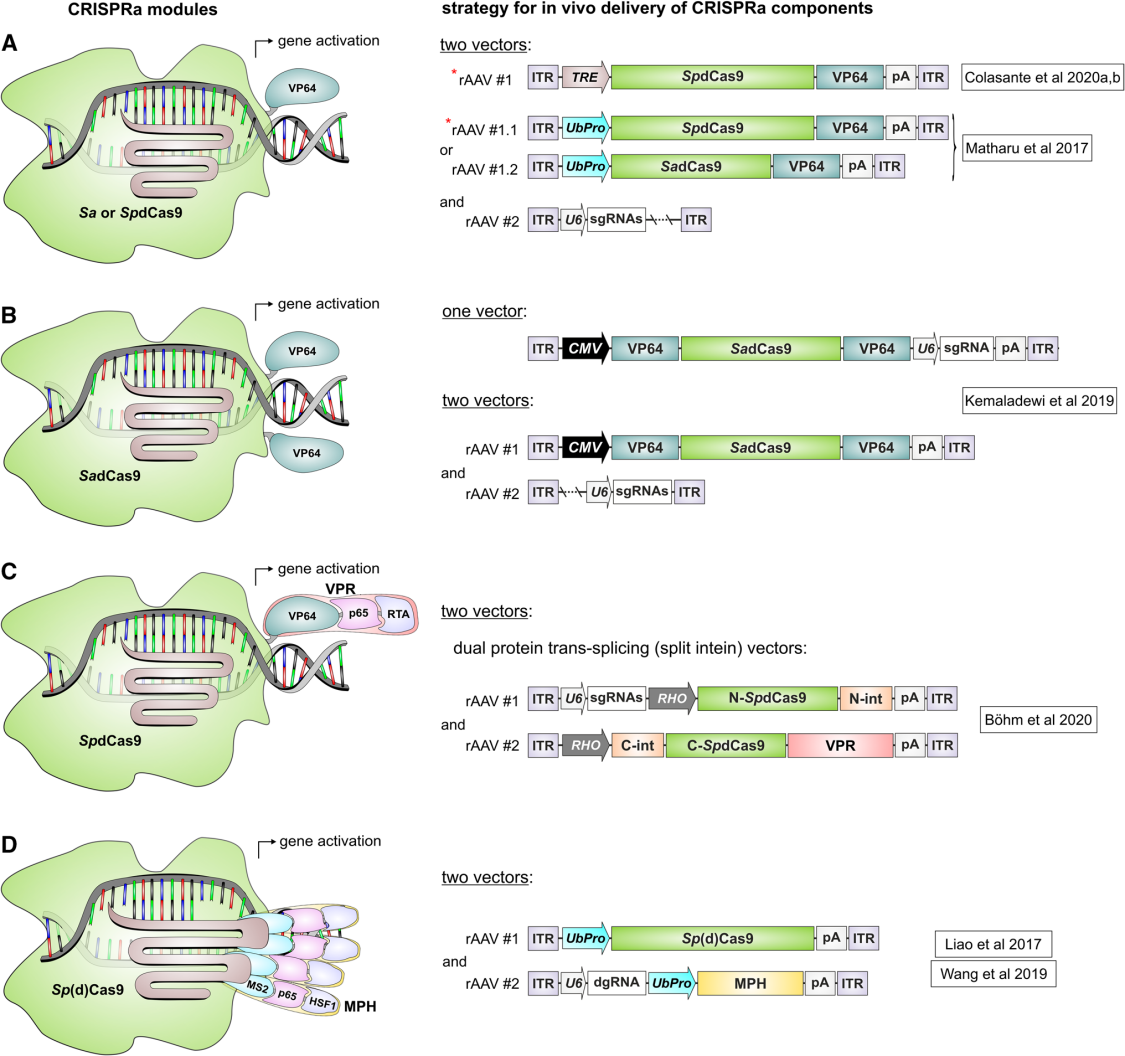
Overview of CRISPRa modules that have been successfully applied to treat genetic or acquired diseases. Right panel, strategies and expression cassettes used for in vivo delivery of the individual modules. The red asterisk highlights the expression cassette which exceeds the packaging capacity of rAAV vectors (4.7 kb). *TRE* tetracycline response element. *UbProm* ubiquitous promoter (e.g., CAG or EF1a). *pA* polyadenylation signal, *U6* human Pol III promoter, *CMV* cytomegalovirus immediate-early promoter, *RHO* human Rhodopsin promoter, *N- or C-SpdCas9* N-or C-terminal part of split *Sp*dCas9, *N-or C-int* N-or C-terminal part of split intein, *dgRNA* “dead” sgRNA with short spacer sequence (14–15 bp) harboring a MS2 stem-loop

The proof-of-concept for programmable gene activation was established well before the CRISPR-Cas era using zinc-finger nucleases (ZFNs) and transcription activator-like effector nucleases (TALENs) fused to trans-activating domains [7]. One advantage of gene activation via ZFNs and TALENs is the fact that these modules do not require dual AAV vectors for in vivo expression due to the small size of these proteins. Nevertheless, ZFNs and TALENs require a design of new protein arrays for each new target sequence in the genome. In comparison, the CRISPRa system allows for a much simpler design, as target specificity can be achieved only by replacing the protospacer sequence within the sgRNA cassette. Another key advantage of CRISPR-Cas-based systems over ZFNs or TALENs is the ease of multiplexing, i.e., editing multiple genes simultaneously using different sgRNAs [8]. The first CRISPRa module described consists of dCas9 fused at its C-terminus to four VP16 transcription activating (trans-activating) domains derived from the herpes simplex virus (dCas9-VP64) [9, 10]. dCas9-VP64 is characterized by low to moderate gene activation efficiency. To increase gene activation and thus expand the range of applications of CRISPRa, more effective CRISPRa modules have been engineered by fusing additional trans-activating domains or their binding sites to dCas9. This has led to the development of several second-generation CRISPRa variants, of which some lead representatives will be described in this review. One important representative is the dCas9-VPR module, in which the NF-κB (nuclear factor kappa-light-chain-enhancer of activated B cells) p65 subunit and R transactivator (Rta) domains from Epstein-Barr virus were fused to dCas9-VP64 [11]. By comparison, the dCas9-SunTag module contains a tag with multiple binding sites for VP64 [12]. In other CRISPRa systems, in addition to fusing trans-activating domains to the Cas protein, the sgRNAs tetraloops and stemloops have been modified to contain binding sites (protein binding RNA aptamers) for other trans-activating molecules. This includes the dCas9-SAM (synergistic activation mediator) module, which consists of dCas9-VP64 and an additional fusion protein containing the coat protein of the RNA bacteriophage MS2, p65, and the Heat Shock Transcription Factor 1 (HSF1). This MS2-p65-HSF1 (MPH) complex can bind to the tetraloops and stemloops of the modified sgRNA via MS2 and facilitate transcription via p65 and HSF1 domains [13]. Recently, different CRISPRa modules were compared side-by-side with the result that dCas9-VPR, dCas9-SAM, and dCas9-SunTag consistently provided the highest gene activation efficiencies between different cell types and species [14]. Comparing only these three most potent modules with each other, the *trans* activation efficiency was largely gene-specific. These modules have been applied in numerous in vitro studies to date, however only a few in vivo studies exist. In the translationally focused in vivo studies to date, CRISPRa has been successfully applied to treat genetic or acquired diseases such as diabetes mellitus, cancer, epilepsy, Dravet syndrome, muscular dystrophy, acute kidney injury, obesity or retinitis pigmentosa [15–22]. In this context, recombinant adeno-associated viral (rAAV) vectors were used to express *Staphylococcus aureus* (*Sa*)dCas9-VP64 [18, 20], *Sp*dCas9-VP64, *Sp*(d)Cas9-MPH, a truncated variant of the dCas9-SAM module lacking the VP64 domain [19], and S*p*dCas9-VPR [15] (Fig. 1). In the study by Matharu et al., two different AAV vectors were used to rescue the obesity phenotype in the heterozygous loss of function Sim1 mouse model by overactivation of the remaining functional allele. The first vector expresses *Sp*Cas9-VP64 or *Sa*dCas9-2xVP64 and the second one the sgRNA cassette. A similar *Sp*Cas9-VP64 dual AAV vector system was used in two recent studies to activate the *Kcn1* or the *Scn1a* gene to treat the respective mouse model for epilepsy or Dravet syndrome [16, 17]. From the dual AAV vector perspective, the main difference from the Mathary et al. study was that *Sp*Cas9-VP64 expression was driven by a doxycycline-dependent TetOn (TRE) promoter (Fig. 1A). Yamagata et al. also established a therapy for Dravet syndrome in the *Scn1a*-haplodeficient mouse model by overactivating the intact endogenous *Scn1a* locus. For this purpose, the authors crossed transgenic mice expressing dCas9-VPR with *Scn1a*-haplodeficient mice and applied only the *Scn1a*-targeting sgRNAs via AAV vectors [22]. Nevertheless, the results from CRISPRa-expressing transgenic mice, although helpful to provide proof-of-concept, are less meaningful from a therapeutic perspective, as such a strategy bypasses CRISPRa delivery, which is one of the major hurdles to translation. In comparison, Kemaledawi et al. either used (i) a single rAAV vector expressing *Sa*dCas9-VP64 and one sgRNA or (ii) two separate AAVs, the first encoding *Sa*dCas9-VP64 and the second expressing three sgRNAs, to activate the *Lama1* gene in a mouse model of congenital muscular dystrophy type 1A (MDC1A). This disease is caused by a mutation in *Lama2* gene, which is functionally related to *Lama1* (Fig. 1B). In another study, Böhm et al. used dual split-intein-based AAV vectors to activate the rhodopsin-related *Opn1mw* (middle wavelength opsin, M-opsin) gene in rod photoreceptors in the rhodopsin-deficient mouse model for retinitis pigmentosa, the most common form of inherited retinal blindness. The first vector contains three sgRNAs and one half of dCas9-VPR fused to a split-intein, and the second one encodes the remaining half of the dCas9-VPR-intein fusion protein. In this approach, the split-inteins are required for scarless reconstitution of *Sp*dCas9-VPR when dual rAAVs are co-administered (Fig. 1C). Split-intein-based dual AAV vectors were also used in another study to express a *Sp*dCas9 CRISPRa version containing VP64 at the C- and N-termini of Cas9 and additionally the p65 domain at the C-terminus. Using this approach, moderate activation of the *Afp* locus was observed in the mouse retina, but the therapeutic potential of this activation was not investigated [23]. In the study by Liao et al., most in vivo experiments were performed in transgenic mice expressing Cas9. These mice were crossed with mouse models for different diseases and injected with an AAV vector expressing gene-specific sgRNAs and MPH to investigate the basic potential of gene activation for the therapy of the respective disorders. In the injected mice, activation of the follistatin (*Fst*), utrophin, or klotho genes was shown to increase the muscle mass or mitigate the muscle wasting phenotype (e.g., Duchenne muscular dystrophy) or, in the case of klotho, to attenuate the disease phenotype of acute kidney injury. In addition, the pancreatic and duodenal homeobox gene 1 (*Pdx1*) was also activated in liver cells to induce the formation of insulin-producing cells, which could represent a new therapeutic option for diabetes mellitus type I. Recently, another study utilized a very similar *Sp*dCas9-MPH-based dual AAV vector strategy for multiplexed activation of endogenous genes to induce an anti-tumor adaptive immunity [21] (Fig. 1D).

Compared to *Sp*dCas9-VPR, *Sp*dCas9-SAM, or *Sp*dCas9-SunTag modules, *Sp*dCas9-VP64 results in substantially lower gene activation efficiencies [14] and is therefore less suitable for the treatment of diseases that require higher expression levels of target genes to achieve a therapeutic effect. Due to its considerably shorter coding sequence (3159 bp) *Sa*Cas9 offers a key advantage over *Sp*Cas9 (4104 bp) in terms of gene delivery using rAAVs, as these gold standard gene therapy vectors have a comparatively low genome packaging capacity (4.7 kb). Accordingly, in contrast to *Sp*(d)Cas9-VP64, *Sa*(d)Cas9-VP64 expression cassettes including all regulatory elements needed for effective expression (e.g., inverted terminal repeats, promoter, polyadenylation signal) typically do not exceed the payload of rAAVs when promoters shorter than 800 bp are used [24].

Given that the most potent VPR, SAM or SunTag-based CRISPRa variants exceed the packaging limit of rAAVs even if using *Sa*Cas9 or other alternatives with similar size such as *Campylobacter jejuni Cj*Cas9, [25], *Geobacillus stearothermophilus Geo*Cas9 [26], or CasX [27] these Cas9 variants thus do not offer a noticeable advantage over *Sp*Cas9 for in vivo expression of these modules. Additionally, compared to *Sp*Cas9, *Sa*Cas9 has a more restrictive protospacer adjacent motif (PAM) sequence (5'-NNGRRT-3' for *Sa*Cas9 vs 5'-NGG-3' for *Sp*Cas9), which limits the number of suitable sgRNAs for activation of specific loci. Nevertheless, many studies have identified Cas variants with less restrictive PAM sequences (e.g. [28, 29]) and the field of identification and optimization of different therapeutically relevant Cas proteins is still very dynamic. Hence, it remains to be seen which of these Cas variants will win the race in the long run.

In addition, gene expression can also be regulated via recruitment of enzymes directly affecting DNA methylation or inducing histone modifications [30]. DNA methylation of specific regions results in gene silencing [31], whereas different histone modifications (e.g., acetylation, methylation, phosphorylation, formylation) lead to gene activation [32]. Several CRISPR-Cas modules exist that can be used for these purposes [33]. Among those, CRISPRa modules developed in the past contain Cas9 linked to demethylases such as dCas9 fused to the catalytic domain of ten-eleven translocation dioxygenase1 (TET1), [34], to catalytic histone acetyltransferase (HAT) core domain of the human E1A-associated protein p300 [35], or to histone methyltransferase PR domain zinc finger protein 9 (PRDM9) [36].

### Advantages of CRISPRa for therapeutic applications

Compared to other existing strategies for the therapy of genetic or acquired diseases, CRISPRa-based approaches offer several important advantages: (i) They are independent of target gene size and can be used in multiplexing experiments to activate different genes simultaneously. (ii) They induce little to no off-target effects in vivo [18–20]. This is partly because potential off-target sequences, in addition to matching the target sequence, must also be part of a promoter or enhancer region. (iii) They can be used for simultaneous activation and knockdown of different genes in target cells. This requires the catalytically active Cas enzyme and the use of sgRNAs with spacer sequences of different lengths. For spacers less than 16 bp in length, Cas loses its catalytic activity, but the CRISPRa module is still capable of gene activation [6, 37]. Such an approach can be used, for example, to treat gain-of-function mutations that require simultaneous knockout of the mutant allele in addition to gene supplementation. (iv) In contrast to CRISPR/Cas modules which rely on the endonuclease activity, CRISPRa generally do not induce permanent changes in the genome. While permanent modification of the target locus is the desired effect in some diseases (e.g., to accomplish efficient gene knockdown), this can also become a problem if such modifications cause undesirable effects (e.g., large deletions) or occur at undesirable locations in the genome [38–42]. (v) In contrast to gene complementation strategies, where only the coding sequence is introduced, gene activation involves all natural processes of gene expression including mRNA splicing or the expression of long non-coding RNAs. (vi) In contrast to CRISPR/Cas-based strategies designed for excision or correction of the disease-causing mutation, CRISPRa approaches are mutation-independent and thus allow the treatment of a much larger number of patients.

### Limitations of the CRISPRa strategy

Despite the important advantages listed above, CRISPRa approaches also have some notable limitations: (i) They require permanent expression of the gene activating module in target cells. Therefore, in contrast to approaches using the Cas endonuclease activity, delivery techniques tailored to short-term transgene expression (e.g., delivery of protein or mRNA by liposomes, virus-like particles, or nanoparticles) are not appropriate for CRISPRa. To date, studies in mouse models have not observed any negative effects on target cells despite prolonged expression of the CRISPRa components [15, 18–20]. Nevertheless, this circumstance needs to be further investigated before using CRISPRa modules in initial non-humane primates or clinical studies. A hit-and-run strategy that enables a long-term effect at the epigenome level has already been developed for the CRISPRi system [43], and it remains to be seen whether a similar approach can be achieved for CRISPRa in a therapeutic context. (ii) Similar to all other CRISPR-Cas systems, there is a risk of an immune reaction against the bacterial Cas protein in target cells [44]. (iii) They require appropriate delivery methods for efficient and long-lasting expression of CRISPRa cassettes in vivo. This is particularly difficult to be achieved for the large expression cassettes exceeding the packaging capacity of rAAV vectors [8]. (iv) When using vectors that are not integrated into the genome of the target cell (e.g., rAAVs), the application of CRISPRa is usually limited to non-diving cells, unless the therapeutic effect can be achieved by short-term expression of the target gene. Nevertheless, usage of AAV and CRISPR/Cas-based strategies is also limited due to the preexisting immunity to both components in the human population [45–50]. This can be partially overcome by developing new AAV vectors capsids [51] or using immune orthogonal AAVs and Cas proteins [52]. (v) The (over)expression of a gene in the target cell can also potentially have cell-damaging effects. In these cases, fine-tuning of the expression of affected genes may be required.

### Strategies for in vivo delivery of CRISPRa modules for therapeutic purposes

Most therapeutic studies currently use rAAV vectors for stable (long-term) expression of transgenes in target cells [53]. Accordingly, despite many differences in the design of the individual vectors, all the studies reporting beneficial effects of the CRISPRa system in a therapeutically relevant experimental setup utilized AAV vectors so far.

The advantages of this method include inter alia high transduction efficiency, low immunogenicity, good tolerability, low risk of genomic integration, long-term expression, etc. [4, 54]. Moreover, just like CRISPR/Cas technology itself, the AAV vectors have already been approved by the FDA. Thus, the combination of both is well suited for translationally oriented studies. However, the limited DNA uptake capacity of these vectors requires alternative solutions for expression of large cassettes. Among the CRISPRa modules that have been successfully evaluated in vivo, *Sa*(d)Cas9-VP64 does not exceed the packaging capacity of rAAVs (Fig. 1A, B). However, due to its restrictive PAM sequence and low gene activation efficiency of VP64 alone, this module is less flexible and less broadly applicable. In comparison, expression of other more effective CRISPRa modules, such as *Sp*dCas9-VPR or *Sp*dCas9-MPH, requires co-application of two different AAV vectors, each encoding only a portion of the entire module (Fig. 1C, D). Such dual AAV vector approaches depend on high co-transduction efficiency of both vectors in target cells. High co-transduction rates (> 90%) were observed after local administration of rAAVs into some organs or tissues, such as the retina or brain [55–58]. From the efficacy perspective of dual AAV vectors, local administration (if possible) should therefore be preferred when dealing with diseases affecting only one or a few tissues or organs. However, local administration is also advantageous from a safety point of view, as fewer global immune reactions or side effects are to be expected compared to systemic application.

The transduction efficiency of dual rAAV vectors after systemic administration is understudied, but it is reasonable to assume that significantly higher AAV vector amounts are necessary to co-transduce a considerable percentage of target cells. However, higher rAAV dosage is associated with higher side effect risks, e.g., immune responses [59]. Therefore, further studies are necessary to investigate the principal suitability and the side effect profile of dual rAAV vectors after systemic administration. Some new strategies, such as co-packaging of multiple AAVs into extracellular vesicles, might help to decrease the dosage of dual AAVs needed for systemic application [60].

Despite their differences in the underlying molecular mechanisms, dual rAAV vectors expressing split genes follow the same principle: after the gene fragments are packaged into two different rAAV vectors and co-applied to the target tissue, they can be reconstituted at one of three levels: genomic, mRNA or protein level [61]. Historically, gene reconstitution at the genome level is the eldest dual rAAV vector strategy. There are several versions of this method and all of them depend on two processes called concatemerization and/or homologous recombination [62, 63]. In recent years another approach aiming at the reconstitution of the split rAAV genome at the protein level attracted increasing attention. In this context, each of the split coding sequences is fused to split inteins, which are small affinity tags capable of autocatalytic and scarless reconstitution of the split fragments at the protein level [64]. Split inteins have been shown to operate at high efficiency in vivo using rAAVs [15, 44, 65–69]. Nevertheless, the efficiency of this method strongly depends on the split site within the protein and relies on the presence of specific residues at the split and the neighboring positions. In addition, the reconstitution efficiency of proteins provided with split inteins also depends on protein folding. Optimal results are only achieved if both separate parts of a protein are folded independently of each other and if the exposed split inteins have good steric access to each other [64, 70]. Therefore, finding an efficient split position for split intein approaches requires an elaborative and time-consuming screening process for each gene. Finally, the split intein approach creates equimolar amounts of potentially immunogenic or pathogenic proteins (inteins), raising safety concerns for translational studies. Such concerns may be even more pronounced if split inteins are combined with Cas9-based approaches, as the co-expression of Cas9 and inteins could lead to additive or even synergistic effects with respect to the host immune responses. Conclusively, there is an unmet need for developing alternative approaches for reconstitution of split genes using dual rAAV vectors. Reconstitution of the split fragments at the mRNA level via a process called mRNA trans-splicing is a largely unexplored option for dual rAAV vectors and it remains to be shown whether this technology can overcome the drawbacks of currently used dual rAAV vector approaches.

Aside from their limited DNA packaging capacity, other factors exist that impede the widespread use of AAVs in clinical trials. One reason is the preexisting immunity to naturally occurring AAVs in the human population [45, 47, 50]. Although many AAV capsid variants have been reported that show a partial immune escape (e.g., [71–82]), their performance has to be further investigated in more advanced preclinical and clinical settings. Another bottleneck for the broad usage of AAVs in clinical trials are their high manufacturing costs, which drives up the cost of such therapies to unimaginable heights that hardly any healthcare system in the world can currently afford [83]. However, increasing competition and the number of production sites, as well as improved production technologies and processes, could lead to a significant reduction in these costs in the near future and thus make such therapies accessible to the broad mass of the population.

Apart from AAVs, to my knowledge, no other strategies have been used to deliver CRISPRa in vivo in a therapeutically relevant setting. This is most likely due to the fact that gene activation requires sustained expression of CRISPRa, which is unlikely to be achieved with non-viral systems, e.g., by delivery of the corresponding protein and sgRNAs using other techniques such as nanoparticles or virus-like particles [8]. In contrast, for other CRISPR-Cas-based systems designed to induce permanent changes in the genome sequence, transient expression is certainly an attractive solution to reduce the risk of adverse effects. Accordingly, several non-viral gene delivery strategies have been used for such purposes with varying success rates [84–92].

Other commonly used virus-based alternatives to AAVs for in vivo gene delivery are lentiviral or adenoviral vectors (LVs or Ad) [93]. An advantage of LVs and Ad is their much higher genome packaging capacity (10 kb and 36–37 kb, respectively) [94, 95]. This allows packaging of all relevant CRISPRa modules and corresponding sgRNA expression cassettes into a single vector. However, compared to AAVs, LVs and Ad also have some important drawbacks. These include the less preferable safety profile of these vectors due to their higher immunogenic activity. In addition, due to their size, Ad and LV are less able to cross biological barriers or diffuse away from the injection site (lateral spreading), which is required for the transduction of a larger area after a single local injection [93]. In addition, LVs carry a higher risk of integrating the transgenes into the host genome (insertional mutagenesis). Integrase-deficient LV variants have also been developed, but these lead to transient transgene expression and are therefore less suitable for CRISPRa approaches [96, 97]. This also applies to Ad variants which inherently show transient transgene expression [94].

### Final remarks and future directions

CRISPRa is a modular and broadly applicable strategy for the therapy of genetic or acquired diseases. The very high therapeutic potential of CRISPRa approaches faces several challenges, mainly related to delivery, long-term expression, and immunity. AAV vectors remain the gold standard for CRISPRa delivery and long-term expression. Their limitations, such as limited cargo capacity or pre-existing immunity in the human population, may be overcome in the future by the development of novel AAV capsid variants with improved tissue tropism, higher packaging capacity, lower immunogenicity, and/or higher immune escape [83]. To minimize the potential negative effects of long-term CRISPRa expression, the use of controllable, i.e., demand-driven, expression systems, such as doxy- or tetracycline-dependent promoters or other inducible expression systems, would be beneficial [98]. However, this requires concomitant administration with the appropriate drugs, all of which may also cause side effects themselves and should therefore be balanced with the expected therapeutic benefit. Another approach that may facilitate gene delivery in the future is the development of new (shorter) CRISPRa variants that can be packaged into a single AAV vector without noticeable sacrifice in flexibility or efficacy. The new CRISPRa variants should meet similar requirements as the new AAV capsids in terms of their immunogenicity and immune escape. In addition to AAV-based approaches, the development of novel viral or non-viral delivery strategies could substantially advance the field and further exploit the potential and capabilities of CRISPRa in a therapeutic setting. However, given the advantages of AAVs, any new approaches will have to compete with these vectors in terms of efficacy and safety, and it remains to be seen whether this ambitious goal can be achieved in the foreseeable future.

Conclusively, although some barriers to gene delivery still need to be overcome and the immunogenicity better investigated, CRISPRa holds great translational potential and its implementation into first clinical trials could benefit millions of patients worldwide.

## Data Availability

Not applicable.
